# Deep learning-based Desikan-Killiany parcellation of the brain using diffusion MRI

**DOI:** 10.1038/s41598-026-54446-8

**Published:** 2026-06-03

**Authors:** Yousef Sadegheih, Dorit Merhof

**Affiliations:** 1 Faculty of Informatics and Data Science, University of Regensburg, Regensburg, 93053 Germany; 2 Fraunhofer Institute for Digital Medicine MEVIS, Bremen, 28359 Germany

**Keywords:** Deep learning, Segmentation, Parcellation, Diffusion MRI, Computational biology and bioinformatics, Engineering, Mathematics and computing, Medical research, Neurology, Neuroscience

## Abstract

Accurate brain parcellation in diffusion MRI (dMRI) space is essential for advanced neuroimaging analyses. However, most existing approaches rely on anatomical MRI for segmentation and inter-modality registration, a process that can introduce errors and limit the versatility of the technique. In this study, we present a novel deep learning-based framework for direct parcellation based on the Desikan-Killiany (DK) atlas using only diffusion MRI-derived data. Our method utilizes a hierarchical, two-stage segmentation network: the first stage performs coarse parcellation into broad brain regions, and the second stage refines the segmentation to delineate more detailed subregions within each coarse category. We conduct an extensive ablation study to evaluate various diffusion-derived parameter maps, identifying a top-performing combination of fractional anisotropy, trace, sphericity, and maximum eigenvalue that enhances parcellation accuracy compared with previously used parameter choices. When evaluated on the Human Connectome Project, our approach achieves higher Dice Similarity Coefficients compared to existing state-of-the-art methods. On the Consortium for Neuropsychiatric Phenomics dataset, where reliable voxel-wise DK reference labels in diffusion space are not available, our method demonstrates label-free evidence of robustness across different image resolutions and acquisition protocols by producing more homogeneous parcellations as measured by the relative standard deviation within regions. This work represents a step toward more practical dMRI-based brain parcellation by avoiding the need for anatomical MRI and subject-specific anatomical-to-diffusion registration at inference time. The implementation of our method is publicly available on https://github.com/xmindflow/DKParcellationdMRI.

## Introduction

Diffusion Magnetic Resonance Imaging (dMRI) is a valuable imaging technique that captures the movement of water molecules within biological tissues, offering indirect yet detailed information about microstructural organization^1^. Unlike traditional anatomical MRI, which focuses on tissue contrast and morphology, dMRI reveals the structure and connectivity of white matter pathways through fiber tracking and tractography^2^. As a result, dMRI has become widely used in both research and clinical settings for investigating brain development, aging, and neurological conditions^3^.

An essential step in analyzing brain imaging data is parcellation, which divides the brain into meaningful anatomical or functional regions. This step provides a framework for regional analysis and supports tasks such as structural connectivity mapping and disease-related abnormality detection^4–8^. In diffusion MRI studies, accurate parcellation is especially important for building reliable connectomes and assessing localized tissue changes^9^.

Traditionally, brain parcellation is performed on high-resolution anatomical MRI (typically T1- or T2-weighted scans) using established software tools such as FreeSurfer (FS)^10^ or CAT12^11^. The anatomical parcellations are then aligned with the diffusion images through inter-modality registration. However, this process is error-prone due to distortions introduced by echo-planar imaging (EPI) used in dMRI^12–14^ and the lower spatial resolution of diffusion data^15^. Misregistration between modalities can lead to inaccurate label assignments and compromise downstream analyses. Furthermore, anatomical MRI may not always be available in certain clinical or research workflows^9^.

To address these practical limitations at inference time, recent studies have developed deep learning methods that perform brain parcellation directly on dMRI data^9,16–22^.

These approaches often use convolutional neural networks (CNNs) to extract features from diffusion-derived maps such as fractional anisotropy (F/FA) and mean diffusivity (MD). Advanced methods like DDParcel^9^ and DDEvENet^22^ have achieved notable performance by combining multiple input features and ensemble learning to improve segmentation quality without relying on anatomical MRI. However, current dMRI-based parcellation methods still face several challenges. Many approaches rely on 2D or 2.5D representations, where networks are trained on individual slices in axial, coronal, or sagittal views. Although this design, seen in FastSurfer^23^, reduces computational load, it limits the ability to fully capture 3D spatial relationships in the brain. This can result in reduced segmentation accuracy, especially in regions with complex anatomy^24^. Fully 3D models have shown improved results in medical image segmentation tasks by learning volumetric features and context^25,26^, which can be beneficial for detailed brain parcellation.

Another challenge lies in the choice of input features for parcellation. Current models use different combinations of diffusion parameters, but there is no clear agreement on which combination offers the best performance. For example, DDParcel^9^ uses FA, MD, and tensor eigenvalues in a multi-branch network, while DDEvENet^22^ employs a confidence-weighted ensemble approach with different tensor eigenvalues. This lack of standardization in feature selection can affect generalization across datasets with varying scanning protocols^27^.

Finally, segmentation models based on the Desikan-Killiany (DK) atlas^28^ face challenges related to imbalanced label distributions. The DK atlas^28^ is a widely used brain parcellation scheme in neuroimaging, particularly for structural and connectivity analyses. It divides the cerebral cortex into 101 regions (including both hemispheres), based on anatomical landmarks derived from gyral and sulcal patterns. These regions vary substantially in size and volume across the brain, which presents a challenge during model training. Larger brain regions tend to dominate the loss function during model training, while smaller regions are underrepresented, leading to poorer segmentation of these finer anatomical structures^27^. This imbalance can hinder tasks that require precise boundary definitions in all brain regions, making it a significant issue for accurate parcellation. Since this work targets DK parcellation in diffusion space, supervised training requires DK-consistent reference labels. We therefore use FreeSurfer-derived DK parcellations projected into diffusion space during model development. After training, however, the model operates directly on diffusion-derived inputs and no longer requires anatomical MRI or subject-specific anatomical-to-diffusion registration at inference time.

In this paper, we present a 3D deep learning framework designed for direct DK parcellation using only diffusion MRI-derived data. Our method is based on a hierarchical, coarse-to-fine segmentation strategy that first predicts larger anatomical divisions and then refines them into detailed parcels. By using fully 3D networks, our model can better learn spatial patterns and boundaries from volumetric data. Additionally, we perform a thorough comparison of diffusion-derived parameters to identify those that contribute most to segmentation performance.

Our main contributions are: ❶ A detailed ablation study on the influence of different diffusion-derived features on parcellation accuracy. ❷ A hierarchical 3D segmentation architecture that improves performance in both large and small brain regions. ❸ Demonstrated improved supervised parcellation performance on a high-quality diffusion MRI dataset and complementary label-free robustness under lower-resolution, heterogeneous acquisition conditions.

Through this work, we aim to provide a reliable and practical solution for brain parcellation directly in the diffusion space, removing the need for anatomical MRI and subject-specific anatomical-to-diffusion registration at inference time. This contributes to more efficient and accessible analysis of diffusion MRI data.

## Datasets and preprocessing

### Datasets

Our evaluation utilized two datasets: the Human Connectome Project (HCP)^29^ and the Consortium for Neuropsychiatric Phenomics (CNP)^30^. For the HCP dataset, we used a subset of 100 young, healthy adults, consisting of 46 males and 54 females, with an average age of 29.1 years. The dataset was split into training, validation, and test sets with a ratio of 50:30:20, respectively. The dMRI data from the HCP dataset have a voxel size of $1.25^3 mm^3$ and include 18 baseline images along with 270 diffusion-weighted images from three b-shells, with b-values of 1000, 2000, and 3000 $s/mm^2$. The structural MRI data (T1w and T2w) for this dataset has a voxel size of $0.7^3 mm^3$.

The CNP dataset consists of 272 young adults, including individuals with various health conditions such as schizophrenia, bipolar disorder, and attention-deficit/hyperactivity disorder (ADHD), as well as healthy controls. However, only 214 participants had the necessary structural and dMRI data for our analysis. The cohort included 114 males and 100 females, with an average age of 33.2 years. Of these, 108 were healthy controls, 41 had bipolar disorder, 36 had ADHD, and 29 had schizophrenia. Each dMRI scan in the CNP dataset has a voxel size of $2^3mm^3$ and contains one baseline image along with 64 diffusion-weighted images with a b-value of 1000 $s/mm^2$. The structural MRI (T1w) data has a voxel size of $1^3mm^3$.

Because the CNP dataset is a legacy single-shell dataset containing diffusion data only at $b=1000~\mathrm {s/mm^2}$, we restricted the present study to the $b=1000~\mathrm {s/mm^2}$ shell in HCP as well. This design ensured compatibility across datasets and remained aligned with prior dMRI-based parcellation studies^9,22^, which also focused on DTI-derived features in the $b=1000~\mathrm {s/mm^2}$ setting.

### Preprocessing

We apply different preprocessing steps for the training and inference phases. For training, we use data from the HCP dataset, which has demonstrated high reliability as pseudo-ground-truth reference labels. The HCP data is preprocessed using the HCP minimal processing pipeline^31^. This pipeline includes motion correction, eddy current correction, EPI distortion correction, and registration to the 6th generation nonlinear MNI152 space^32^. A brain mask is also extracted during this process. Prior studies have shown that FreeSurfer parcellation obtained from anatomical MRI and projected to diffusion space can provide a practical set of reference labels for supervised DK parcellation in dMRI space, particularly when high-quality data such as HCP are used^9,20^.

**Fig. 1:**
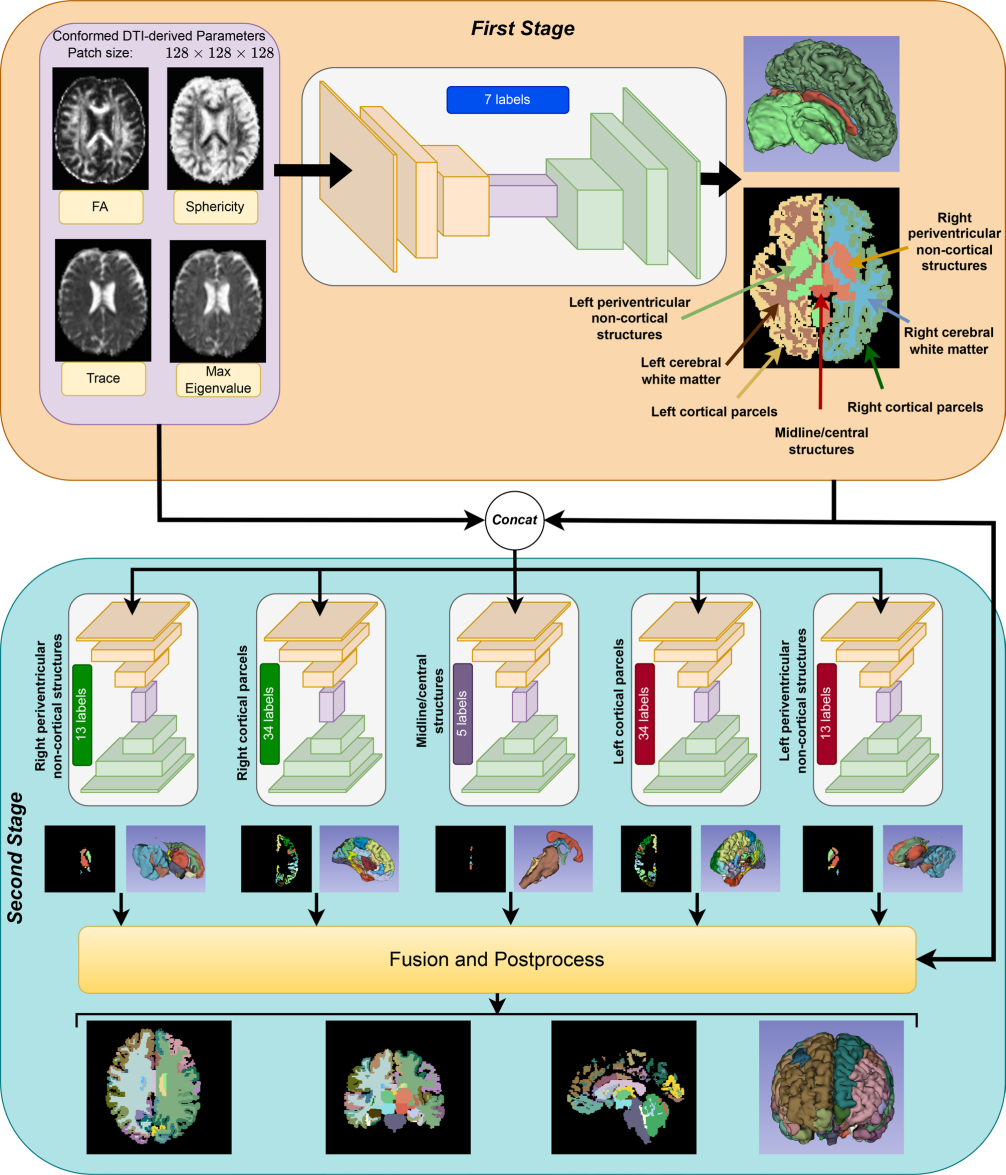
Hierarchical two-stage framework overview.

We use the FreeSurfer white-matter parcellation (wmparc) generated from the structural MRI data and convert it into the 101-label DK label set used in this study by an explicit remapping procedure. This conversion includes a small number of label merges and remappings needed to align the wmparc output with the DK-based label definition adopted here. A complete and reproducible mapping table is provided in the Supplementary Material. This approach is justified because the HCP pipeline performs extensive postprocessing on white matter parcellation to ensure accurate identification of anatomical regions. Having a well-coregistered parcellation in dMRI space allows us to perform parcellation similarly to a typical segmentation task.

For the CNP data, the dMRI data is not preprocessed in the same way as HCP data. Therefore, we apply a validated preprocessing pipeline^33^ that includes eddy current correction and motion correction using FSL tools^34^, along with brain mask extraction^35,36^. Susceptibility-induced EPI distortion correction was not applied, because the auxiliary data required by the preprocessing workflow used here were not available in the CNP release used in this study.

Following established protocols^9,35,37^, we obtain parcellation in the dMRI baseline space by nonlinearly coregistering the FS parcellation to the dMRI space using ANTs^38^. The parcellation is then resampled to the dMRI space using nearest-neighbor interpolation. This coregistration and parcellation serve as the baseline reference for the parcellation task.

After preprocessing the Diffusion-Weighted Imaging (DWI) data, we extract the necessary diffusion parameters. These include FA, trace, sphericity^39^, and the maximum eigenvalue maps (For a detailed discussion on the rationale behind the selection of these parameters, refer to Section Parameter Impact Study). We use 3D Slicer^40^ with the SlicerDMRI extension^41^ to fit the diffusion tensor model from the DWI data using a least squares method. Then, the required parameter maps are calculated using SlicerDMRI.

To account for head motion during MRI acquisition, all derived parameter maps are transformed to the nonlinear 6th generation MNI152 space^32^. Since FS performs parcellation in a standardized conformed space, we apply this conformation to the parameter maps as well. This ensures all maps are resampled to a uniform spacing of $1\times 1\times 1\ \textrm{mm}^3$, a resolution of $256^3$, and Left-to-right, Inferior-to-superior, Anterior-to-posterior (LIA) orientation, which matches FS’s output.

Throughout the pipeline, all data and derived parameters remain co-registered in this conformed MNI space. After parcellation prediction, the results are transformed back to the original native space to align with the original DWI data. We perform these transformations using the baseline (b = 0) image as the reference. First, the transformation from native space to MNI space is calculated. Then, the derived parameters are moved to MNI space using this transformation. Finally, the inverse transform is applied to return the parcellation results to native space. Our model training uses the nnUNet framework, which applies the same preprocessing steps for all MRI-based inputs, including the derived diffusion parameter maps.

## Methodology

The proposed training strategy enhances segmentation accuracy by leveraging the hierarchical structure of brain parcellation. It consists of two stages: first, a coarse segmentation (Section Coarse Parcellation) partitions the brain into broad regions, and then, a refinement step within each region (Section Fine Parcellation) enables the delineation of finer anatomical parcels. This two-stage approach is designed to improve label assignment to dMRI data, with each stage optimized for its specific task to improve overall segmentation quality. The two stages are trained sequentially rather than jointly: the coarse model is optimized first, followed by the fine subnetworks for the individual coarse compartments.

It is important to highlight that our methodology includes a preprocessing step, which was detailed in Section Preprocessing. In this section, we focus on the processing and post-processing (Section Post-processing) procedures. A visual representation of the overall workflow is provided in Fig. 1. While the framework is compatible with any 3D model, the selection of the model plays a crucial role in determining the final performance. Stronger, more robust segmentation models generally yield better results.

### Coarse parcellation

In the coarse parcellation stage, a single 3D model is used for segmentation; however, it does not directly predict all 101 labels of the DK atlas. To reduce the severe class imbalance of the original 101-label DK segmentation problem, we grouped the final labels into seven anatomically interpretable coarse categories: (1) left cerebral white matter, (2) right cerebral white matter, (3) left deep/periventricular non-cortical structures, (4) right deep/periventricular non-cortical structures, (5) left cortical parcels, (6) right cortical parcels, and (7) midline/central structures. The grouping preserves hemispheric organization while separating cortical, white-matter, deep gray/periventricular, and midline compartments, which differ substantially in morphology, size, and diffusion-derived appearance. The resulting partition contains $1+1+13+13+34+34+5=101$ final labels. A complete mapping from the final FreeSurfer labels to the seven coarse categories is provided in Supplementary Table 1.

This grouping strategy was designed not only to improve anatomical interpretability, but also to alleviate the extreme label imbalance of the original single-stage formulation. We quantified this effect by aggregating voxel counts over the training set and summarizing imbalance using the maximum-to-median voxel-count ratio. In the original 101-label formulation, this ratio was 43.6, indicating that the largest label was far larger than a typical label. After regrouping into the seven coarse categories, the ratio decreased to 1.2, showing that the largest coarse class was only modestly larger than the median coarse class.

The model input consists of 3D patches derived from four dMRI-based parameter maps: fractional anisotropy, trace, sphericity^39^, and maximum eigenvalue. These maps are spatially aligned and resampled to a consistent isotropic resolution of 1mm^3^, conforming to the LIA orientation and a volume size of 256^3^. The model’s output is a segmentation map consisting of the seven predefined categories.

### Fine parcellation (sub-region segmentation)

In the fine parcellation stage, only those coarse categories that contain further anatomical subdivision are refined. Specifically, five dedicated segmentation networks are trained for the left cortical group, right cortical group, midline/central group, left deep/periventricular non-cortical group, and right deep/periventricular non-cortical group. The left and right cerebral white matter labels are not omitted from the final parcellation; rather, they are predicted in the first stage and directly retained in the final 101-label output. We did not refine them further because, in the label definition used in this work, they are already final labels and are not subdivided into additional white-matter subregions.

This staged design was chosen deliberately rather than training the entire hierarchy end-to-end. In practice, joint training would require simultaneous backpropagation through the coarse model and multiple 3D fine subnetworks, substantially increasing GPU memory demands and complicating optimization. By training the stages sequentially, we retain stable 3D patch-based training, while allowing the coarse stage to learn global anatomical localization and the fine subnetworks to focus on within-compartment refinement.

Each network receives as input the same four dMRI-derived parameter maps (Section Coarse Parcellation) and the coarse segmentation masks. These inputs are combined into a five-channel volume, where four channels correspond to the dMRI parameter maps and one channel represents the coarse segmentation mask. This mask is normalized to a range between 0 and 1, ensuring that label values from the coarse segmentation do not disproportionately influence the input scale.

As illustrated in Fig. 1, these five networks produce segmented regions corresponding to cortical areas (divided into 34 labels), central regions (five labels), and the remaining regions (13 labels per hemisphere). The outputs from these independent networks are then combined to generate the complete fine parcellation, which provides a detailed segmentation of the brain into 99 sub-regions.

### Post-processing

Following the fine segmentation, all generated labels are merged to form the complete set of 101 parcellation labels. These labels are then transformed back from the conformed space used during preprocessing to the original dMRI space using the inverse transformation from the preprocessing stage.

In the post-processing step, the coarse segmentation serves as a mask, restricting the fine segmentations to their corresponding coarse regions. This ensures that each subregion is confined within the boundaries defined by the coarse parcellation. Any subregion that falls outside its corresponding coarse segmentation mask is removed. A dilation step is then applied to the remaining fine segmentations to improve boundary continuity and reduce small gaps between adjacent regions.

To further refine the segmentation, we apply 3D connected-component analysis using 26-connectivity for each non-background DK label. Since each anatomical label is expected to represent a single continuous entity, connected components are ranked by size, and only the largest connected component is retained for each label. No additional minimum voxel-count threshold is used. This step removes small disconnected components that may arise from segmentation artifacts or noise, ensuring that isolated regions are excluded from the final parcellation while preserving the principal anatomical structure of each label.

Subsequently, the combined labels are resampled to match the original dMRI resolution using nearest-neighbor interpolation, implemented in 3D Slicer^40^. This resampling produces the final parcellation aligned with the original dMRI data. Finally, the labels are mapped to the corresponding FS lookup table labels.

## Experimental setup and metrics

### Experimental setup

The pipeline and framework were implemented using modules from the nnUNet framework^25^ while using PyTorch 2.1.0^42^. Each stage of the framework was trained with a batch size of 2 and a patch size of $128^3$. For training, each stage used a single NVIDIA A100 GPU (80 GB VRAM). The learning rate settings followed the original hyperparameters outlined in the related work. Specifically, for the U-Net^25^ architecture, we used the SGD optimizer with Nesterov momentum (0.99) and a weight decay of $3\times 10^{-5}$. The learning rate followed a polynomial decay strategy (power 0.9) with an initial value of 0.01. For the MedNeXt^43^ model, we used the AdamW optimizer^44^ with an initial learning rate of 0.001 and applied the same polynomial decay strategy. For SwinUNETR^45^, we used the same learning rate as the nnUNet.

The training process used the default nnUNet objective, namely a composite loss consisting of Dice loss^46^ and cross-entropy loss with equal weighting (1:1). Unless otherwise stated, the cross-entropy term was not class-weighted. Data augmentation techniques followed those described in^25,47,48^. Training was carried out for 1000 epochs, with 250 iterations per epoch, leading to a total of 250,000 iterations.

To generate the final prediction for each stage, we used sliding-window inference with 0.5 overlap along each spatial dimension and a Gaussian weighting kernel to reduce boundary effects between adjacent windows. Training time varied depending on the architecture and the number of labels predicted at each stage. On average, each stage of the U-Net version required 25 GPU hours, while the MedNeXt and SwinUNETR versions required approximately 40 and 35 GPU hours, respectively. For clarity, nnUNet, MedNeXt-M-K3, and SwinUNETR are treated as the corresponding single-stage baselines. The broader computational trade-off is summarized in Supplementary Table 6, which reports both the total training cost of the hierarchical framework, computed as the sum of the coarse model and all five fine subnetworks, and the corresponding inference times.

**Table 1:** Diffusion-derived parameter ablation study using 2D nnUNet (F: FA, T: Trace, S: Sphericity, P: Planarity, L: Linearity, E1: Max eigenvalue, E2: Mid eigenvalue, E3: Min eigenvalue). Italic and bold indicate the best and second-best results in each modality section, respectively. Results are reported as DSC with standard deviation in parentheses.

	Param.		DSC		Param.		DSC		Param.		DSC		Param.	DSC
1 Modality	F		74.15 (0.05)		T		73.16 (0.01)		L		71.93 (0.05)		P	69.97 (0.08)
	S		74.34 (0.08)		E1		**74.39 (0.06)**		E2		73.51 (0.12)		E3	*75.01 (0.09)*

	Param.	DSC	Param.	DSC	Param.	DSC	Param.	DSC	Param.	DSC	Param.	DSC	Param.	DSC
2 Modality	F+T	*76.34 (0.05)*	F+L	74.37 (0.09)	F+P	74.44 (0.08)	F+S	74.72 (0.17)	T+L	75.40 (0.13)	T+P	74.89 (0.12)	T+S	76.26 (0.07)
	L+P	74.45 (0.06)	L+S	74.69 (0.09)	E1+E2	75.52 (0.05)	E1+E3	76.27 (0.04)	E2+E3	75.70 (0.07)	F+E1	76.28 (0.10)	F+E2	**76.33 (0.07)**
	F+E3	76.28 (0.15)	T+E1	75.90 (0.03)	T+E2	75.16 (0.06)	T+E3	76.12 (0.09)	S+E1	76.32 (0.02)	S+E2	76.26 (0.08)	S+E3	76.29 (0.07)

	Param.		DSC	Param.		DSC	Param.		DSC	Param.		DSC	Param.	DSC
3 Modality	E1+E2+E3		76.28 (0.05)	F+E1+E3		76.35 (0.14)	T+E1+E3		76.15 (0.08)	S+E1+E3		76.32 (0.20)	T+S+E3	76.23 (0.11)
	T+S+E1		76.36 (0.07)	T+S+F		*76.48 (0.10)*	T+F+E1		**76.41 (0.02)**	T+F+E3		76.37 (0.09)		
4 Modality	F+T+E2+E3		76.39 (0.15)	T+S+E1+E3		76.16 (0.15)	T+F+S+E3		**76.49 (0.10)**	T+F+S+E1		*76.52 (0.08)*	T+F+E1+E3	76.46 (0.09)

	Param.		DSC		Param.		DSC		Param.		DSC		Param.	DSC
>4 Modality	T+F+E1+E2+E3		76.42 (0.07)		T+S+E1+E2+E3		76.37 (0.06)		T+F+S+E1+E3		*76.47 (0.06)*		T+F+S+E1+E2+E3	**76.41 (0.07)**

### Evaluation metric

For evaluation on the HCP test data, where diffusion-space reference parcellations are available, we use the Dice Similarity Coefficient (DSC) and the 95th percentile Hausdorff Distance (HD95) as the primary supervised metrics. In addition, to provide a common homogeneity-based criterion across datasets, we also compute the Relative Standard Deviation (RSD), also known as the Coefficient of Variation (CV), for diffusion-derived measures within each parcellated region. Lower RSD values indicate greater within-region homogeneity.

For the CNP data, where comparable voxel-wise reference labels are not available, RSD serves as the primary label-free evaluation criterion. On HCP, RSD is interpreted as a complementary metric rather than a replacement for DSC and HD95, since the DK atlas is anatomically defined and the models are trained directly on projected DK reference labels. The RSD for each region is defined as:

$$RSD = \frac{\textrm{std}}{\textrm{mean}}$$

Previous studies in diffusion MRI^9,49,50^ have shown that lower RSD values indicate greater homogeneity within regions, which reflects better parcellation quality. Statistical significance of the HCP DSC improvements was assessed using planned pairwise Welch two-sample t-tests between each standalone model and its corresponding framework-enhanced version, followed by Bonferroni correction across the three architecture-level comparisons. For the CNP dataset, patient-wise RSD values were compared separately for FA, MD, and sphericity using one-way repeated-measures ANOVA across methods, followed by Bonferroni-corrected paired post hoc comparisons against T1w-reg.

## Results and discussion

**Table 2:** Results of the test set of the HCP dataset compared to the SOTA architectures. Italic and bold indicate the best and second-best results, respectively. Values are reported as the mean with the standard deviation in parentheses.

Dimension	Model	DSC $\uparrow$	HD95 $\downarrow$
2.5 D	FastSurfer^23^	75.57 (0.14)	2.571 (0.008)
	DDParcel^9^	78.96 (0.11)	1.682 (0.013)
	DDEvENet^22^	78.55 (0.12)	1.684 (0.011)
General 3D	nnUNet^25^	79.64 (0.09)	1.523 (0.007)
	SwinUNETR^45^	79.05 (0.03)	1.575 (0.004)
	MedNeXt-M-K3^43^	**81.35 (0.10)**	**1.474 (0.010)**
	LHU-Net^51^	74.66 (0.03)	1.903 (0.010)
	UNETR^52^	76.17 (0.06)	1.752 (0.020)
	UNETR++^48^	75.80 (0.03)	1.778 (0.018)
	CoTR^53^	77.60 (0.11)	1.608 (0.010)
	nnFormer^47^	74.60 (0.10)	1.800 (0.011)
	SegFormer3D^54^	71.72 (0.56)	2.082 (0.080)
	SwinUNETR-V2^55^	78.85 (0.12)	1.579 (0.011)
	TransBTS^56^	76.43 (0.04)	1.667 (0.038)
Ours 3D	$OURS_{unet}$	81.12 (0.06)	*1.469 (0.009)*
	$OURS_{MedNeXt}$	*82.09 (0.05)*	**1.474 (0.011)**
	$OURS_{SwinUNETR}$	79.92 (0.03)	1.573 (0.005)

### Parameter impact study

Table 1 presents the results of the parameter impact study, which investigates how different parameters derived from Diffusion Tensor Imaging (DTI) data contribute to the DK parcellation task when using a deep learning approach. The motivation for this analysis stems from the variety of parameters employed in existing literature^9,22^ and the recognition that a thorough modality impact study is critical for deep learning-based segmentation tasks. As some parameters may overlap or introduce redundancy into the network, understanding the individual and combined effects of these parameters is essential for optimizing model performance^27^.

For this study, we selected the 2D nnUNet architecture^25^ as an efficient screening model for comparing a large number of parameter combinations. To verify that the observed trends were not specific to the 2D setting, we additionally performed the corresponding ablation using 3D nnUNet; the full 3D results and the corresponding statistical analysis are provided in the Supplementary Material (Supplementary Note 2 and Supplementary Table 3).

The parameters used in our modality impact study include the eigenvalues from the DTI data: maximum eigenvalue (E1), mid eigenvalue (E2), and minimum eigenvalue (E3). From these eigenvalues, several derived parameters can be calculated, such as Trace (the sum of the eigenvalues), FA, Linearity (L/CL), Planarity (P/CP), and Sphericity (S/CS). Specifically, FA is computed using the formula:

$$FA = \sqrt{\frac{(E_1 - E_2)^2 + (E_2 - E_3)^2 + (E_1 - E_3)^2}{2(E_1^2 + E_2^2 + E_3^2)}}$$

and the other parameters are similarly derived from the eigenvalues:

$$CL = \frac{E_1 - E_2}{E_1 + E_2 + E_3}, \quad CP = \frac{2(E_2 - E_3)}{E_1 + E_2 + E_3}, \quad CS = \frac{3E_3}{E_1 + E_2 + E_3}$$

**Fig. 2:**
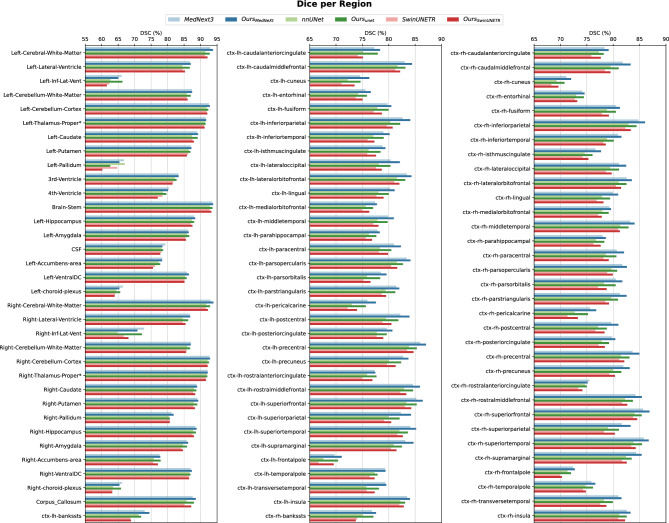
Detailed DSC for each region of the FS DK parcellation.

Our results in Table 1 indicate that diffusion-derived parameters each provide distinct contributions to cortical and subcortical parcellation when individually used as input to a convolutional neural network. The minimum eigenvalue (E3) stands out by producing the highest DSC among all single-parameter inputs. This outcome suggests that E3 is particularly sensitive to microstructural differences that are vital for accurately separating anatomical regions. This can be explained by E3’s close relationship with radial diffusivity, reflecting the degree of restriction experienced by water molecules as they move perpendicular to the dominant orientation of cellular structures. Such sensitivity is especially helpful in the cortex and subcortex, where subtle differences in the arrangement and packing of cells define many boundaries. A likely reason for E3’s superiority over E1 is its higher specificity for non-white matter regions. E1, which reflects axial diffusivity, is often elevated in both white matter and cerebrospinal fluid, thereby reducing its ability to pinpoint boundaries between gray and white matter or between brain tissue and fluid-filled spaces. In contrast, E3 tends to be lower in restricted tissues and higher in more isotropic environments, allowing it to better highlight regional changes in tissue properties. As a result, E3 provides the neural network with clearer signals for distinguishing both cortical microstructure and regions near the ventricles. Shape-based parameters such as CL and CP show limited effectiveness when used alone. Their relatively low performance suggests that while these measures reflect certain geometric aspects of water diffusion, they do not offer sufficient contrast or specificity to allow reliable identification of anatomical boundaries. Interestingly, the sphericity parameter (CS), which measures how close diffusion is to being the same in all directions, performs better than other shape-based metrics. This is notable, as sphericity is not typically emphasized in previous studies, but our results indicate that it can add meaningful information about tissue organization, possibly by highlighting areas with more isotropic diffusion, such as certain gray matter regions or transition zones at tissue interfaces. Notably, E3 achieved the highest DSC among the single-parameter inputs in the 2D screening study, and this same trend was confirmed by the supplementary 3D validation. This consistency suggests that the minimum eigenvalue captures particularly useful information for distinguishing cortical and subcortical structures in the proposed parcellation setting.

**Fig. 3:**
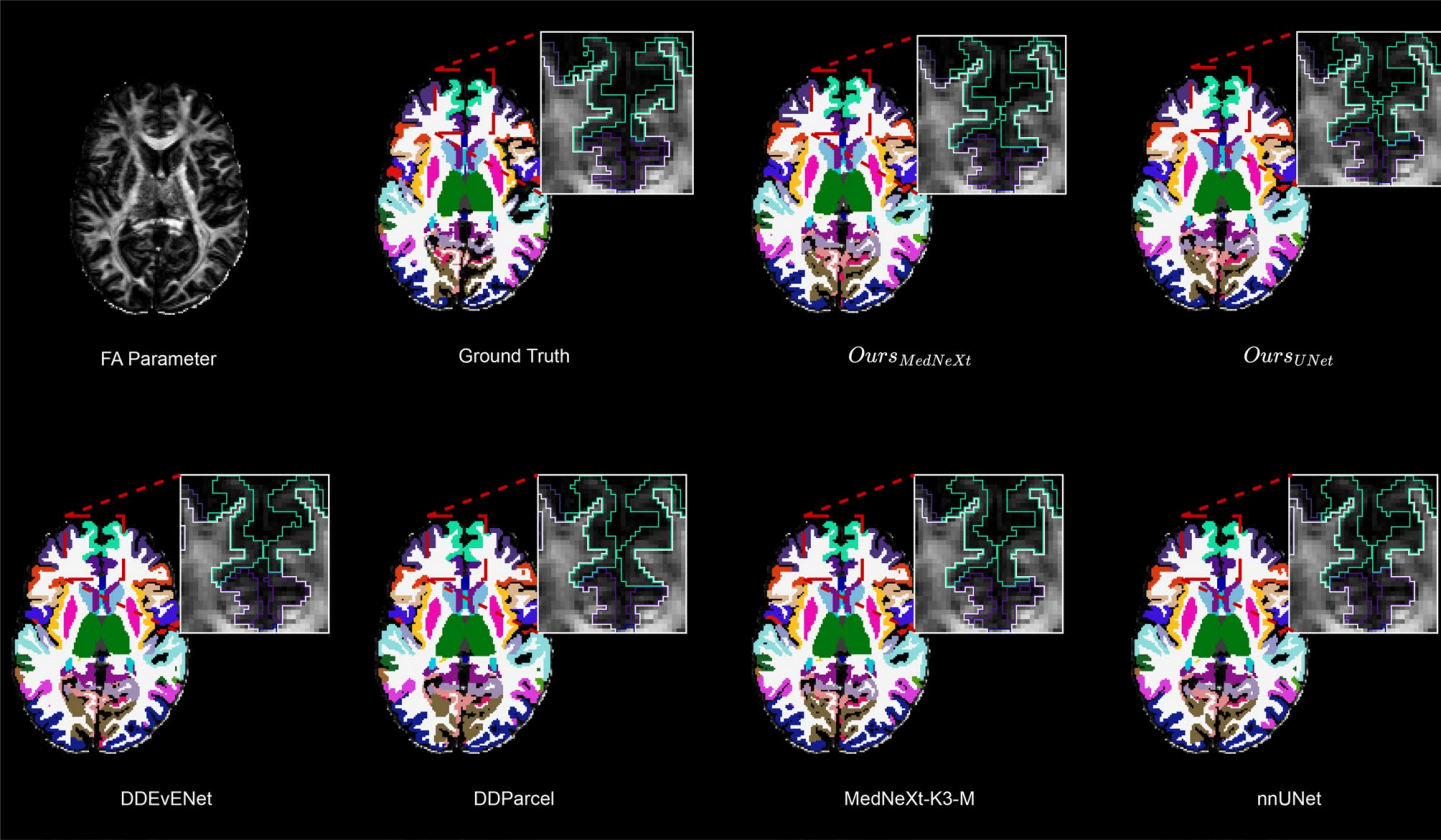
Comparison of parcellations from SOTA models and our proposed framework on the HCP test set.

When examining dual-parameter combinations, we observe further differences in performance and synergy. Although Trace alone yields only moderate results, its combination with FA achieves the highest DSC among all two-parameter inputs. This finding indicates that Trace and FA capture distinct, yet complementary, aspects of tissue structure: Trace measures the total amount of diffusion, while FA quantifies how directional the diffusion is. The network thus benefits from receiving information about both the strength and the pattern of diffusion, allowing for more precise parcellation.

Additionally, combining Trace with CS leads to improved results that are comparable to those achieved with the Trace-FA pair. This can be understood by examining the relationship between the shape metrics FA and S. Both FA and S measure how diffusion deviates from isotropy, but in opposite ways: FA is high when diffusion is strongly directional, while S is high when diffusion is nearly equal in all directions. Our analysis reveals a strong negative correlation between FA and S, meaning they largely provide overlapping information. This is confirmed by our findings that pairing FA and S without Trace does not improve model performance. In some cases, such combinations may even be counterproductive, as overlapping features reinforce the same signals instead of adding new insight. However, when either shape metric is combined with Trace, the network benefits from having access to both shape and overall diffusion magnitude, which results in better parcellation. These results highlight the importance of selecting input parameters that are not only informative but also non-redundant, as combining measures that capture different aspects of tissue properties is key for effective parcellation.

The analysis of three-parameter combinations further emphasizes the value of complementary information. The set of Trace, S, and FA results in a clear increase in mean DSC, suggesting that including both magnitude (Trace) and shape (FA, S) features enables the network to capture a wider range of tissue characteristics. Notably, the relationship between FA and S becomes more useful when Trace is present, as regions with high Trace and low FA, such as the choroid plexus, may benefit from the additional contrast provided by sphericity. This pattern supports the view that a combination of directional, magnitude, and isotropy information can improve parcellation performance.

An important observation is that although all scalar diffusion metrics are derived from the three eigenvalues of the diffusion tensor, using the raw eigenvalues directly as model input does not lead to better performance than using carefully constructed metrics such as FA, Trace, and CS. This may reflect the fact that summary metrics provide the network with features that are already aligned with biologically and anatomically meaningful properties, reducing the burden on the network to learn these relationships from scratch. In practice, this is especially important when the available training data is limited, or when trying to segment complex brain regions where contrasts are subtle and context-specific.

Further experiments reveal that a four-parameter input consisting of Trace, CS, FA, and the maximum eigenvalue (E1) achieves the highest mean DSC of all tested configurations. To statistically assess this parameter choice, we compared $T+F+S+E1$ with the second- and third-best configurations in Table 1, namely $T+F+S+E3$ and $T+F+S$, using two-sided paired *t*-tests followed by Bonferroni correction. These comparisons showed that the differences among the top three configurations were not statistically significant after correction ($p_{\textrm{Bonf}}>0.05$). Therefore, we do not interpret $T+F+S+E1$ as statistically superior to the other top-ranked combinations, but rather as the highest mean-performing member of a statistically comparable top-performing group.

We further compared $T+F+S+E1$ with parameter configurations corresponding to previously used DK parcellation input choices in the literature^9,22^, including $F+T+E2+E3$ and $F+T+E1+E2+E3$, as well as the best compatible single-parameter baseline *E*3. In contrast to the comparisons within the top-performing group, $T+F+S+E1$ showed significantly higher DSC than these literature-based or baseline configurations after correction. The detailed corrected *p*-values and the corresponding 3D validation results are provided in Supplementary Note 2. These results support the use of $T+F+S+E1$ for the subsequent experiments, while also indicating that several closely related combinations form a statistically comparable high-performing set.

The addition of E1 likely introduces information about the dominant direction of diffusion, which can help the network distinguish between tissues with similar overall diffusivity but differing in fiber orientation. This is particularly relevant for differentiating cortical regions from subcortical structures, where directional features are more pronounced. However, performance does not continue to improve when all available diffusion parameters are included. In fact, it starts to decline, probably due to overparameterization. Providing too many input channels, especially those that are redundant or noisy, can dilute the gradients that are important for learning, reduce network generalization, and even introduce confusion for the model during training. These findings suggest a trade-off: while adding informative, non-overlapping features can help, including excessive or highly correlated parameters may hurt model performance.

Finally, our work challenges the common practice, seen in prior studies such as^9,22^, of relying solely on FA as the main input for diffusion MRI-based parcellation. While FA remains a strong and widely adopted parameter, our results make clear that it does not, by itself, yield the best possible segmentation performance. Other parameters, especially E3, as well as combinations involving Trace and CS, bring valuable, and in some cases unique, information that can improve model accuracy. Therefore, it is important for future studies to carefully consider which inputs to use, focusing on both the individual value of each parameter and the way they interact. In particular, choosing parameters that provide different, rather than overlapping, information appears to be essential for effective and generalizable parcellation pipelines.

In conclusion, the parameter impact study demonstrates the importance of systematic, data-driven input selection for diffusion MRI-based DK parcellation. The selected $T+F+S+E1$ configuration achieved the highest mean DSC and significantly outperformed previously used or compatible baseline parameter configurations in both the 2D screening experiment and the supplementary 3D validation. However, it was not statistically superior to the other closely ranked top configurations after Bonferroni correction. Therefore, we interpret $T+F+S+E1$ as an empirically strong and statistically supported input choice relative to prior parameter configurations, while acknowledging that several related combinations based on Trace, FA, sphericity, and eigenvalue-derived information may provide comparable performance. Overall, these findings suggest that effective dMRI-based DK parcellation benefits from combining complementary diffusion information, including diffusion magnitude, directional anisotropy, isotropic diffusion characteristics, and dominant-direction diffusivity.

**Table 3:** RSD results of FA, MD, and Sphericity on the CNP dataset. Values are reported as the mean with the standard deviation in parentheses.

	$OURS_{unet}$	$OURS_{MedNeXt}$	$OURS_{SwinUNETR}$	T1w-reg
$RSD_{FA}$	0.479 (0.031)	0.489 (0.028)	0.485 (0.030)	0.634 (0.038)
$RSD_{MD}$	0.250 (0.024)	0.245 (0.024)	0.242 (0.024)	0.353 (0.030)
$RSD_{S}$	0.179 (0.019)	0.180 (0.020)	0.177 (0.019)	0.239 (0.028)

### Model and framework

Table 2 summarizes the performance of various 2.5D and 3D models tested on the HCP dataset. For general-purpose architectures that support multi-channel volumetric inputs, we used the top-performing four-parameter combination identified in our parameter impact study, namely $T+F+S+E1$, in order to enable a common-input comparison across models. However, some baselines are inherently tied to a more restrictive or method-specific input design. In particular, FastSurfer^23^ accepts only a single input modality. Based on the results from our parameter impact study, we therefore used the minimum eigenvalue (*E*3), which showed the strongest single-parameter performance, as the most favorable compatible input for FastSurfer. For the methods presented in^9,22^, we adhered to their original published input parameter configurations, namely $F+T+E1+E2+E3$ and $F+T+E2+E3$, respectively, because these modality definitions are part of the way those methods were proposed and evaluated. Replacing them with $T+F+S+E1$ would require method-specific architectural adaptation and further hyperparameter optimization, and would therefore no longer represent a faithful reproduction of the published baselines. Accordingly, Table 2 should be interpreted as a common-input comparison for general-purpose architectures and as a faithful published-setting comparison for input-constrained or task-specific baselines.

From the results shown in Table 2, it is evident that 3D CNN-based models perform well in terms of parcellation accuracy. However, hybrid and transformer-based 3D models generally demonstrate lower performance compared to the CNN-based models. In particular, more efficient networks, such as SegFormer3D, failed to achieve accurate segmentation, resulting in poorer performance than 2D models.

Our training strategy focused on two CNN-based models, nnUNet^25^ and MedNeXt-M-K3^43^, which both ranked highly in the general 3D architecture category in Table 2. After applying our training approach, the nnUNet model exhibited an improvement of 1.4% in DSC and a reduction in HD95. Similarly, the MedNeXt-M-K3 model saw a 0.74% increase in DSC, achieving a DSC greater than 82%. These results indicate that, when trained with our strategy within the 3D framework, general-purpose 3D models outperform some models specifically designed for the DK parcellation task. Additionally, the 2.5D models, which were created specifically for this task, performed less effectively than the optimized 3D models.

Interestingly, the standalone SwinUNETR model underperformed compared to nnUNet. However, when integrated into our framework, SwinUNETR showed a notable improvement of 0.87% in DSC, surpassing the performance of the standalone nnUNet model. This finding further highlights the benefits of our focused training strategy, which allows general models to achieve high-quality results comparable to specialized models.

To further assess the statistical significance of the observed HCP improvements, we performed planned pairwise Welch two-sample t-tests comparing the DSC scores of each standalone model with those of its corresponding framework-enhanced version across independent runs. To account for multiple comparisons across the three architecture-level tests, Bonferroni correction was applied. All three comparisons remained statistically significant after correction: nnUNet vs. $OURS_{unet}$ ($p_{\textrm{Bonf}}=1.63\times 10^{-4}$), SwinUNETR vs. $OURS_{SwinUNETR}$ ($p_{\textrm{Bonf}}=1.10\times 10^{-5}$), and MedNeXt-M-K3 vs. $OURS_{MedNeXt}$ ($p_{\textrm{Bonf}}=4.68\times 10^{-3}$). An omnibus ANOVA across the six in-house HCP conditions was also significant ($F=945.99$, $p<0.0001$), further supporting the overall difference among methods.

To provide a common homogeneity-based comparison across datasets, we additionally computed HCP RSD and report the results in Supplementary Note 5. In contrast to DSC and HD95, the HCP RSD differences relative to the reference labels were not statistically significant after Bonferroni correction. This is expected because the models are trained directly to reproduce the projected DK reference parcellations in HCP, which encourages the predicted partitions to remain close to the same target definition. As a result, homogeneity is less discriminative than overlap-based accuracy for the HCP setting.

Because label imbalance is inherent to DK parcellation, we additionally evaluated whether replacing the default unweighted cross-entropy term with a class-weighted cross-entropy term could improve performance. In this control experiment, the architecture, input configuration, and training protocol were kept unchanged, and the class weights were computed from the training set using inverse label frequency. The weighted-loss variant did not improve performance; instead, it reduced the validation DSC of the nnUNet architecture from 79.64 (0.09) to 72.15 (0.06). This result suggests that, in our setting, simple loss reweighting is not as effective for addressing class imbalance as the proposed hierarchical coarse-to-fine formulation.

A more detailed comparison of DSC scores for each region in the DK parcellation is provided in Figure 2, comparing the performance of three standalone models against their performance within our dedicated framework. It is important to note that this comparison isolates the effect of the framework design rather than the loss formulation, since both the standalone models and their corresponding hierarchical versions were trained using the same unweighted Dice + cross-entropy objective. The region-wise DSC values show that the benefits of the proposed framework are not limited to large structures such as the left and right cerebral white matter, but also extend to several smaller anatomical regions. This supports the view that segmenting all labels in a one-go formulation can disadvantage smaller and more complex structures, whereas the proposed coarse-to-fine decomposition enables the model to focus more effectively on anatomically related subproblems. For example, improvements are also observed in smaller structures such as the left and right caudate, indicating that the hierarchical strategy benefits not only dominant labels but also underrepresented regions of the DK atlas.

To further assess prediction reliability, we conducted a post-hoc uncertainty analysis based on the softmax outputs. The results, including confidence, uncertainty ($1 - \text {confidence}$), and margin maps, are provided in Supplementary Figure 1. The analysis shows that uncertainty is primarily localized near anatomical boundaries, with our method exhibiting sharper and more confident predictions compared to the baseline.

To assess the practical cost-benefit trade-off of the hierarchical design, we additionally compared the total training time and per-case inference time of each standalone backbone with its corresponding two-stage version. As expected, the hierarchical formulation increases computational cost, but this increase is accompanied by improved segmentation accuracy. Detailed timing and compute results are reported in Supplementary Table 6.

Additionally, Fig. 3 presents qualitative examples of the parcellations generated by the models within our framework alongside those from their standalone counterparts. These visual comparisons further demonstrate that applying a focused training strategy to general 3D models results in higher-quality dMRI parcellation outcomes.

In conclusion, our framework successfully enhances the performance of various models by providing focused, task-specific training, which improves segmentation accuracy in dMRI-based brain parcellation. This approach highlights the potential of leveraging general models with tailored training strategies to achieve state-of-the-art results in neuroimaging tasks.

### External robustness evaluation

Table 3 presents the RSD results on the CNP dataset. We compare the parcellations generated by co-registering T1-weighted FreeSurfer parcellations to dMRI space (T1w-reg) with those produced by the nnUNet, MedNeXt-M-K3, and SwinUNETR models embedded within our framework. The RSD values were calculated for three diffusion-derived measures: FA, MD, and sphericity. Since reliable voxel-wise DK reference labels in diffusion space are not available for CNP, supervised metrics such as DSC and HD95 cannot be computed meaningfully. Therefore, RSD is used as a label-free homogeneity criterion to assess whether each parcellated region contains internally consistent diffusion-derived measurements.

The proposed models consistently achieve lower RSD values than T1w-reg across all three diffusion-derived measures, indicating greater within-region homogeneity. These findings suggest that the framework produces more homogeneous parcellations under the lower-resolution and legacy single-shell acquisition conditions of CNP. However, because CNP lacks voxel-wise diffusion-space reference labels, these results should be interpreted as label-free evidence of external robustness rather than as a direct supervised measure of anatomical accuracy.

The absence of susceptibility-induced EPI distortion correction is important for interpreting the RSD-based evaluation. Residual EPI distortions can spatially displace diffusion signals and affect diffusion-tensor-derived quantities^12,57^. Therefore, EPI correction, if available, would likely influence both the T1w-reg baseline and the proposed dMRI-based models. For T1w-reg, correction could improve anatomical-to-diffusion alignment before projecting FreeSurfer labels into dMRI space. For the proposed models, correction could also affect the predicted parcellations because the input maps, including FA, Trace, sphericity, and eigenvalue-derived parameters, would be computed from the corrected diffusion data. This point is particularly relevant because the proposed models were trained on HCP data processed with EPI distortion correction, whereas the CNP data used here did not include this correction. Applying comparable correction to CNP could therefore reduce the preprocessing-domain mismatch between training and external evaluation. Accordingly, the CNP RSD results should be interpreted as a label-free homogeneity assessment under the available legacy dMRI preprocessing conditions. EPI correction may reduce RSD by improving spatial consistency and reducing tissue mixing within parcellated regions. However, because susceptibility correction can also change the diffusion-derived measures themselves, including FA and MD^58,59^, the exact direction and magnitude of its effect on RSD cannot be determined without corrected CNP data.

To address the difference between supervised HCP evaluation and label-free CNP evaluation, we additionally computed RSD on the HCP test set using the same definition as in the CNP analysis for FA, MD, and sphericity (Supplementary Note 5). This provides a common homogeneity-based criterion across datasets. However, the interpretation of RSD differs between HCP and CNP. On HCP, the models are trained to reproduce the projected DK reference labels, and the predicted parcellations are therefore expected to follow the regional definition of the supervisory labels. Consequently, RSD is less discriminative than DSC and HD95 in this supervised setting, because it primarily reflects whether the predicted partitions preserve the homogeneity profile of the reference parcellation. In contrast, CNP does not provide reliable voxel-wise DK reference labels in diffusion space; therefore, RSD serves as the primary available label-free criterion for assessing within-region homogeneity. The higher RSD of the CNP T1w-reg baseline compared with the HCP reference labels further suggests that these CNP registrations should be interpreted as a baseline rather than as reliable supervision. Thus, the CNP results support external robustness under heterogeneous acquisition conditions, but do not replace supervised cross-dataset validation.

This distinction defines the boundary of the current evaluation framework. Supervised voxel-wise accuracy can be assessed on HCP, where diffusion-space DK reference labels are available. On CNP, the evaluation is limited to label-free homogeneity because comparable reference labels are unavailable. Therefore, the HCP and CNP analyses provide complementary evidence: supervised segmentation accuracy on HCP and external homogeneity-based robustness on CNP. They do not, however, constitute full supervised cross-dataset validation.

To further assess whether the observed trend holds across diagnostic subgroups, we additionally report subgroup-wise CNP RSD results in the Supplementary Material (Supplementary Table 4). Across healthy controls, schizophrenia, bipolar disorder, and ADHD, the proposed models consistently achieve lower RSD than T1w-reg for FA, MD, and sphericity. These findings suggest that the observed homogeneity improvement is not restricted to healthy controls and provide additional label-free evidence of robustness across the heterogeneous clinical subgroups represented in CNP.

To formally test whether the lower CNP RSD values were statistically significant, we performed separate one-way repeated-measures ANOVAs for FA, MD, and sphericity using patient-wise RSD values across the four methods (T1w-reg, OURS$_{unet}$, OURS$_{MedNeXt}$, and OURS$_{SwinUNETR}$). Significant omnibus method effects were observed for FA ($F(3,639)=3408.61$, $p<0.0001$), MD ($F(3,639)=3282.97$, $p<0.0001$), and sphericity ($F(3,639)=1569.91$, $p<0.0001$). Bonferroni-corrected paired post hoc comparisons further showed that each proposed model achieved significantly lower RSD than T1w-reg for all three metrics.

**Fig. 4:**
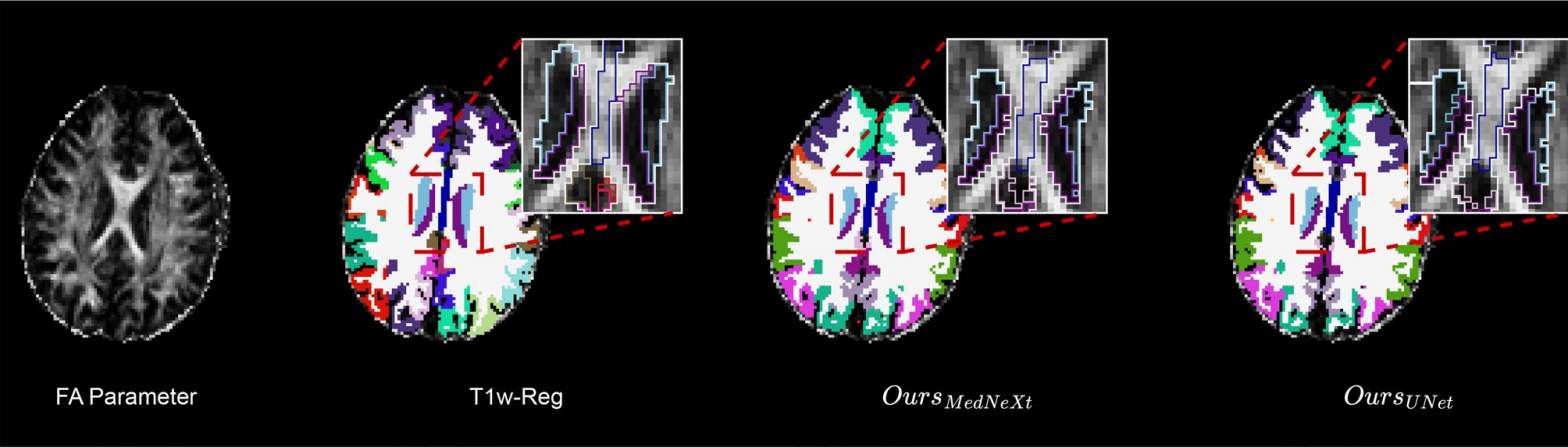
Parcellation comparison between our framework and the co-registered T1-weighted parcellation projected onto the dMRI space (T1w-reg) in the CNP dataset.

Figure 4 presents visual comparisons of the parcellations produced by the proposed models and those obtained through T1w-reg. The qualitative results are consistent with the quantitative RSD findings, showing that the proposed framework produces more spatially coherent parcellations in the available CNP dMRI space. Overall, the CNP evaluation provides label-free evidence of robustness across heterogeneous acquisition conditions. Nevertheless, because RSD measures within-region homogeneity rather than voxel-wise anatomical agreement, these results should be viewed as complementary to the supervised HCP evaluation rather than as a substitute for supervised cross-dataset validation.

## Limitations and future work

While our framework demonstrates promising robustness across datasets and acquisition protocols, several limitations remain. First, the model is trained on the HCP dataset, which primarily consists of young, healthy adults. Although the subgroup-wise CNP analysis suggests consistent behavior across healthy controls, schizophrenia, bipolar disorder, and ADHD, this evaluation is necessarily indirect because CNP does not provide reliable voxel-wise DK reference labels in diffusion space. As a result, supervised metrics such as Dice and HD95 cannot be computed on CNP, and the analysis in these populations is restricted to homogeneity-based evaluation rather than direct supervised segmentation accuracy. Thus, the CNP results provide label-free evidence of external robustness, but they do not constitute full supervised cross-dataset validation. Although RSD provides a common homogeneity criterion across HCP and CNP, it does not directly measure voxel-wise anatomical agreement. On HCP, RSD is complementary because the models are trained to reproduce the projected DK reference labels and therefore tend to preserve the homogeneity profile of the supervisory parcellation. On CNP, RSD is useful as a label-free criterion, but it cannot replace supervised validation. Future work should evaluate the framework on external datasets with reliable diffusion-space DK labels or expert-validated annotations, ideally with harmonized preprocessing and controlled resolution or protocol analyses.

Second, susceptibility-induced EPI distortion correction could not be applied to the CNP dataset because the required auxiliary acquisitions were unavailable. This may affect the absolute RSD values for both T1w-reg and the proposed models. For T1w-reg, residual distortion can influence anatomical-to-diffusion registration. For the proposed models, it can alter the diffusion-derived input maps and increase the preprocessing-domain difference relative to the distortion-corrected HCP training data. Future validation on datasets with the auxiliary data required for EPI correction is needed to directly assess the effect of EPI correction on RSD, anatomical alignment, and model generalization.

Third, although the hierarchical design improves parcellation accuracy, inference time could be further optimized for clinical workflows. Fourth, the present study is restricted to DTI-derived inputs from the $b=1000~\mathrm {s/mm^2}$ shell to remain compatible with legacy single-shell datasets such as CNP. Future work should examine robustness to higher *b*-value shells and multi-shell diffusion modeling, as well as richer microstructural representations such as NODDI or DKI.

## Conclusion

In this work, we have introduced a novel two-stage hierarchical deep learning framework for DK brain parcellation directly from diffusion MRI-derived data. By employing fully three-dimensional network architectures with a coarse-to-fine segmentation strategy, our framework effectively captures the volumetric spatial context and supports more accurate delineation of complex anatomical structures in diffusion space. The framework improves parcellation performance on the HCP dataset and provides a practical approach for predicting DK labels from diffusion-derived parameter maps. Our comprehensive parameter study highlights the value of selecting complementary diffusion-derived parameters, with fractional anisotropy, trace, sphericity, and maximum eigenvalue providing a strong input configuration that improves performance compared with previously used parameter choices. On the CNP dataset, where reliable voxel-wise diffusion-space DK reference labels are not available, the lower RSD values provide label-free evidence that the proposed framework produces more homogeneous parcellations under heterogeneous acquisition conditions, while remaining complementary to the supervised evaluation on HCP. Overall, the proposed method represents a step toward practical dMRI-based brain parcellation by avoiding the need for anatomical MRI and subject-specific anatomical-to-diffusion registration at inference time.

## Supplementary Information

Supplementary Information.

## Data Availability

The diffusion MRI and structural MRI data used in this study are publicly available. Human Connectome Project (HCP) data can be accessed at https://www.humanconnectome.org, and Consortium for Neuropsychiatric Phenomics (CNP) data are available through the OpenNeuro repository under accession number ds000030 and can be accessed at https://openneuro.org/datasets/ds000030/versions/00016. The source code and trained model implementation for the proposed framework are publicly available at https://github.com/xmindflow/DKParcellationdMRI. All data supporting the findings of this study are described within the manuscript

## Code availability

The implementation of our method is publicly available on github.com/xmindflow/DKParcellationdMRI.
