# Supplementary material for: Deep learning-based Desikan-Killiany parcellation of the brain using diffusion MRI
Source: Sci Rep. 2026 Jun 3;16:17182. doi: 10.1038/s41598-026-54446-8 (PMC13234126; doi:10.1038/s41598-026-54446-8)
Supplement: Supplementary file 1 — Supplementary Information. [file 41598_2026_54446_MOESM1_ESM.pdf]

Deep Learning-Based Desikan-Killiany Parcellation of the Brain Using Diffusion MRI

Yousef Sadegheih[1] **and Dorit Merhof**[1,2,*]

[1]Faculty of Informatics and Data Science, University of Regensburg, Regensburg, 93053, Germany
[2]Fraunhofer Institute for Digital Medicine MEVIS, Bremen 28359, Germany
[*]dorit.merhof@ur.de

ABSTRACT

Accurate brain parcellation in diffusion MRI (dMRI) space is essential for advanced neuroimaging analyses. However, most existing approaches rely on anatomical MRI for segmentation and inter-modality registration, a process that can introduce errors and limit the versatility of the technique. In this study, we present a novel deep learning-based framework for direct parcellation based on the Desikan-Killiany (DK) atlas using only diffusion MRI-derived data. Our method utilizes a hierarchical, two-stage segmentation network: the first stage performs coarse parcellation into broad brain regions, and the second stage refines the segmentation to delineate more detailed subregions within each coarse category. We conduct an extensive ablation study to evaluate various diffusion-derived parameter maps, identifying a top-performing combination of fractional anisotropy, trace, sphericity, and maximum eigenvalue that enhances parcellation accuracy compared with previously used parameter choices. When evaluated on the Human Connectome Project, our approach achieves higher Dice Similarity Coefficients compared to existing state-of-the-art methods. On the Consortium for Neuropsychiatric Phenomics dataset, where reliable voxel-wise DK reference labels in diffusion space are not available, our method demonstrates label-free evidence of robustness across different image resolutions and acquisition protocols by producing more homogeneous parcellations as measured by the relative standard deviation within regions. This work represents a step toward more practical dMRI-based brain parcellation by avoiding the need for anatomical MRI and subject-specific anatomical-to-diffusion registration at inference time. The implementation of our method is publicly available on github.com/xmindflow/DKParcellationdMRI.

Supplementary Note 1: Coarse-group definition and class-balance analysis

This supplementary note provides the exact mapping from the final FreeSurfer/DK labels used in this study to the seven coarse categories employed in the first stage of the hierarchical framework. It also reports the class-balance analysis used to quantify the effect of coarse grouping. Voxel-count imbalance was summarized over the training set using the maximum-to-median voxel-count ratio, where a lower value indicates a more balanced label distribution.

Supplementary Table 1. Mapping from final FreeSurfer/DK labels to the seven coarse categories used in the first stage.

Coarse category	FreeSurfer labels included	Number of final labels
Left cerebral white matter	2	1
Right cerebral white matter	41	1
Left deep/periventricular non-cortical structures	4, 5, 7, 8, 10–13, 17, 18, 26, 28, 31	13
Right deep/periventricular non-cortical structures	43, 44, 46, 47, 49–54, 58, 60, 63	13
Left cortical parcels	1001–1003, 1005–1035	34
Right cortical parcels	2001–2003, 2005–2035	34
Midline/central structures	14, 15, 16, 24, 192	5
**Total**		**101**

Supplementary Table 2. Class-balance statistics computed from voxel counts aggregated over the training set. Imbalance is summarized by the maximum-to-median voxel-count ratio.

Segmentation formulation	Number of classes	Max-to-median voxel-count ratio
Original single-stage formulation	101	43.6
Coarse first-stage formulation	7	1.2

Supplementary Note 2: Statistical validation and 3D validation of the parameter impact study

To verify that the conclusions of the parameter impact study were not specific to the 2D screening model, we repeated the ablation using 3D nnUNet. The full 3D results are reported in Supplementary Table 3. Overall, the 3D ablation showed the same trend as the 2D screening study: the highest mean-performing configurations were based on complementary combinations of Trace, FA, sphericity, and eigenvalue-derived information. In absolute terms, the 3D nnUNet achieved an average DSC improvement of 3.45 percentage points over the corresponding 2D nnUNet configurations in this parameter-ablation experiment.

We additionally performed statistical comparisons to support the interpretation of the parameter-selection results. For the 2D screening study reported in Table 1 of the main manuscript, the highest mean-performing configuration was $T+F+S+E1$. We compared this configuration with the second- and third-best configurations, namely $T+F+S+E3$ and $T+F+S$, using two-sided paired t-tests followed by Bonferroni correction across the two top-tier comparisons. The differences among these top-ranked configurations were not statistically significant after correction ($p_{\text{Bonf}} > 0.05$). Therefore, $T+F+S+E1$ should not be interpreted as statistically superior to the other closely ranked top-performing combinations, but rather as the highest mean-performing member of a statistically comparable high-performing group.

To further contextualize the selected input configuration, we compared $T+F+S+E1$ with previously used or compatible baseline parameter configurations. Specifically, we compared it with the best single-parameter input $E3$, the DDEvENet-related configuration $F+T+E2+E3$, and the DDParcel-related configuration $F+T+E1+E2+E3$. Bonferroni correction was applied across these three planned comparisons. In the 2D screening study, $T+F+S+E1$ significantly outperformed $E3$ ($p_{\text{Bonf}} = 6.5\times10^{-5}$), $F+T+E2+E3$ ($p_{\text{Bonf}} = 3.24\times10^{-2}$), and $F+T+E1+E2+E3$ ($p_{\text{Bonf}} = 4.27\times10^{-3}$).

We performed the same statistical analysis for the 3D nnUNet ablation. As in the 2D screening study, the differences between $T+F+S+E1$ and the other top-performing configurations were not statistically significant after Bonferroni correction ($p_{\text{Bonf}} > 0.05$), again indicating that the top-ranked combinations form a statistically comparable high-performing group rather than a single uniquely superior input set.

In contrast, $T+F+S+E1$ significantly outperformed the previously used or compatible baseline configurations in the 3D ablation. Specifically, it achieved significantly higher DSC than $E3$ ($p_{\text{Bonf}} = 2.14\times10^{-4}$), $F+T+E2+E3$ ($p_{\text{Bonf}} = 2.39\times10^{-2}$), and $F+T+E1+E2+E3$ ($p_{\text{Bonf}} = 2.12\times10^{-2}$), after Bonferroni correction across the three planned comparisons. These findings support the selection of $T+F+S+E1$ for the subsequent experiments, while clarifying that several closely related top-ranked configurations provide statistically comparable performance.

Supplementary Table 3. Diffusion-derived parameter ablation study using 3D nnUNet (F: FA, T: Trace, S: Sphericity, P: Planarity, L: Linearity, E1: Max eigenvalue, E2: Mid eigenvalue, E3: Min eigenvalue). Blue and red indicate the best and second-best results in each modality section, respectively. Results are reported as DSC with standard deviation in parentheses.

	Param.	DSC	Param.	DSC	Param.	DSC	Param.	DSC
1 Modality	F	78.14 (0.08)	T	78.18 (0.15)	L	77.16 (0.05)	P	76.57 (0.08)
	S	77.93 (0.08)	E1	78.51 (0.13)	E2	78.17 (0.09)	E3	78.74 (0.09)

	Param.	DSC	Param.	DSC	Param.	DSC	Param.	DSC	Param.	DSC	Param.	DSC	Param.	DSC
2 Modality	F+T	79.37 (0.08)	F+L	78.15 (0.05)	F+P	78.48 (0.13)	F+S	78.52 (0.10)	T+L	78.66 (0.06)	T+P	78.53 (0.08)	T+S	79.27 (0.18)
	L+P	78.64 (0.03)	L+S	78.60 (0.07)	E1+E2	78.01 (0.08)	E1+E3	79.28 (0.07)	E2+E3	78.94 (0.06)	F+E1	79.29 (0.03)	F+E2	79.32 (0.12)
	F+E3	79.30 (0.13)	T+E1	78.99 (0.21)	T+E2	78.47 (0.11)	T+E3	79.06 (0.13)	S+E1	79.31 (0.30)	S+E2	79.27 (0.05)	S+E3	79.26 (0.03)

	Param.	DSC	Param.	DSC	Param.	DSC	Param.	DSC	Param.	DSC
3 Modality	E1+E2+E3	79.31 (0.17)	F+E1+E3	79.39 (0.21)	T+E1+E3	79.04 (0.18)	S+E1+E3	79.40 (0.03)	T+S+E3	79.35 (0.06)
	T+S+E1	79.24 (0.10)	T+S+F	79.58 (0.12)	T+F+E1	79.49 (0.20)	T+F+E3	79.43 (0.01)		
4 Modality	F+T+E2+E3	79.55 (0.12)	T+S+E1+E3	79.43 (0.11)	T+F+S+E3	79.60 (0.08)	T+F+S+E1	79.64 (0.09)	T+F+E1+E3	79.51 (0.11)

	Param.	DSC	Param.	DSC	Param.	DSC	Param.	DSC
>4 Modality	T+F+E1+E2+E3	79.55 (0.09)	T+S+E1+E2+E3	79.50 (0.09)	T+F+S+E1+E3	79.58 (0.10)	T+F+S+E1+E2+E3	79.56 (0.20)

Supplementary Note 3: Subgroup-wise evaluation on the CNP cohort

To assess whether the CNP results were consistent across diagnostic subgroups, we evaluated the relative standard deviation (RSD) separately for healthy controls, schizophrenia, bipolar disorder, and ADHD. Because the CNP dataset does not provide reference DK parcellations in diffusion space, this subgroup analysis is based on the unsupervised homogeneity criterion used in the main manuscript rather than supervised metrics such as Dice or HD95. Lower RSD indicates greater within-region homogeneity and therefore better parcellation quality.

As shown in Supplementary Table 4, all three models embedded within our framework achieved lower RSD than T1w-reg across all four subgroups for FA, MD, and sphericity. These results suggest that the improved within-region homogeneity of the proposed framework is not limited to healthy controls, but remains stable across the pathological cohorts represented in the CNP dataset.

Supplementary Table 4. Subgroup-wise RSD results on the CNP dataset for healthy controls and pathological cohorts. Lower values indicate greater within-region homogeneity and therefore better parcellation quality. Values are reported as mean (standard deviation).

Cohort	Metric	$OURS_{unet}$	$OURS_{MedNeXt}$	$OURS_{SwinUNETR}$	T1w-reg
Control	FA	0.476 (0.036)	0.486 (0.033)	0.480 (0.033)	0.632 (0.045)
	MD	0.248 (0.023)	0.244 (0.023)	0.241 (0.022)	0.355 (0.031)
	Sphericity	0.180 (0.016)	0.182 (0.017)	0.178 (0.015)	0.244 (0.028)
Schizophrenia	FA	0.492 (0.023)	0.498 (0.021)	0.494 (0.022)	0.635 (0.027)
	MD	0.256 (0.023)	0.250 (0.022)	0.248 (0.024)	0.350 (0.026)
	Sphericity	0.174 (0.011)	0.176 (0.010)	0.172 (0.011)	0.229 (0.021)
Bipolar disorder	FA	0.481 (0.023)	0.490 (0.025)	0.490 (0.026)	0.635 (0.028)
	MD	0.251 (0.027)	0.245 (0.028)	0.245 (0.027)	0.352 (0.029)
	Sphericity	0.181 (0.032)	0.183 (0.032)	0.179 (0.033)	0.234 (0.034)
ADHD	FA	0.477 (0.028)	0.487 (0.020)	0.485 (0.026)	0.639 (0.031)
	MD	0.247 (0.024)	0.242 (0.023)	0.239 (0.023)	0.351 (0.029)
	Sphericity	0.176 (0.013)	0.176 (0.012)	0.174 (0.011)	0.235 (0.024)

Supplementary Note 4: Conversion from FreeSurfer `wmparc` to the DK label set

The training labels used in this study were derived from the FreeSurfer white-matter parcellation (`wmparc`) and converted into the 101-label DK label set used by our framework. This conversion was performed through an explicit remapping procedure designed to align the `wmparc` output with the DK-based label definition adopted in this work. The remapping includes a small number of label merges, background assignments, and range-based mappings for white-matter parcellation labels. The full mapping is listed in Supplementary Table 5 to ensure reproducibility.

Supplementary Note 5: Cross-dataset homogeneity-based consistency analysis

We additionally computed the relative standard deviation (RSD) on the HCP test set using the same definition as in the CNP analysis. Specifically, for each parcellation, we calculated the within-region RSD of FA, MD, and sphericity and then averaged these values across all DK regions. This analysis provides a common label-free homogeneity criterion across HCP and CNP, complementing the supervised DSC and HD95 metrics available for HCP.

The primary motivation for using RSD in this study is that the CNP dataset does not provide reliable voxel-wise reference labels in diffusion space. In that setting, supervised metrics such as DSC and HD95 cannot be computed meaningfully, and a label-free homogeneity-based measure is required. We therefore also report RSD on HCP so that the same homogeneity criterion can be examined on both datasets.

At the same time, the HCP analysis highlights an important limitation of RSD in a supervised setting. On HCP, the models are trained directly to reproduce the projected DK reference parcellations. Because the supervision target is anatomically defined and the training objective explicitly encourages agreement with these projected DK labels, the predicted parcellations are expected to remain close to the same regional definition used by the reference. As a result, homogeneity-based measures such as RSD are less discriminative on HCP than supervised overlap-based metrics such as DSC and HD95.

Consistent with this interpretation, on the HCP test set the mean FA-based RSD was 0.496 (0.014) for OURS_{unet}, 0.503

Supplementary Table 5. Explicit remapping rules used to convert the FreeSurfer `wmparc` output into the DK label set used in this study.

Original FreeSurfer label(s)	Mapped label	Rationale
77, 80	removed / background	Hypointensity-related labels not retained in the final DK label set
85	0	Optic chiasm mapped to background
30	2	Vessel label mapped to left cerebral white matter
62	41	Vessel label mapped to right cerebral white matter
72	24	5th ventricle mapped to CSF
3000–3999	2	Left FreeSurfer white-matter parcellation range mapped to left cerebral white matter
4000–4999	41	Right FreeSurfer white-matter parcellation range mapped to right cerebral white matter
251–255	192	Corpus callosum subdivisions merged into the corpus callosum label used in our final set

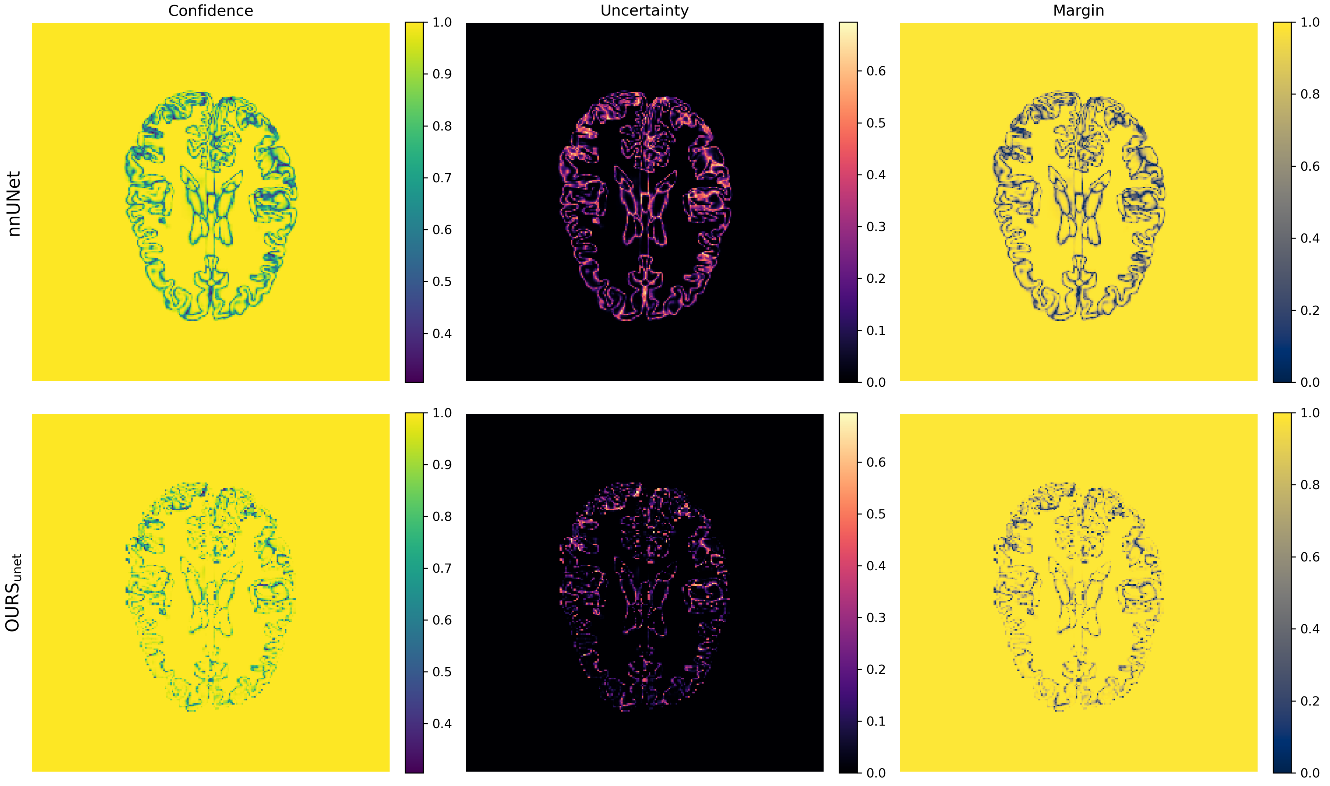

Supplementary Figure 1. Voxel-wise uncertainty analysis for nnUNet (top) and the proposed method (bottom). Columns show confidence (maximum probability), uncertainty ($1-$confidence), and margin (difference between top two probabilities). Our method exhibits higher confidence, lower uncertainty, and sharper transitions in margins.

(0.014) for OURS$_{MedNeXt}$, 0.493 (0.013) for OURS$_{SwinUNETR}$, and 0.503 (0.018) for the HCP reference labels. For MD, the corresponding RSD values were 0.235 (0.018), 0.241 (0.018), 0.232 (0.016), and 0.263 (0.013), respectively. For sphericity, the corresponding RSD values were 0.175 (0.005), 0.177 (0.005), 0.174 (0.005), and 0.217 (0.009), respectively. These results show that the predicted parcellations have RSD values close to, or lower than, those of the HCP reference labels. However, lower RSD on HCP should not be interpreted as a replacement for supervised accuracy, because RSD measures within-region homogeneity rather than voxel-wise anatomical agreement. Therefore, DSC and HD95 remain the primary evaluation metrics on HCP.

In contrast, CNP does not provide reliable diffusion-space DK reference labels. The T1w-reg parcellations in CNP are therefore used as a baseline rather than as ground-truth supervision. Their higher RSD values compared with the proposed models, as reported in the main manuscript, indicate that these registrations are less homogeneous under the available CNP preprocessing conditions. This supports our interpretation that using the CNP T1w-reg labels as a supervisory signal would risk training on less homogeneous and potentially registration-affected labels. Therefore, the CNP analysis is better interpreted as a label-free external robustness assessment rather than as supervised cross-dataset validation.

For this reason, RSD should be interpreted differently in the two datasets. On HCP, RSD is a complementary homogeneity metric because reliable supervised reference labels are available and DSC/HD95 remain the primary measures of segmentation accuracy. On CNP, where comparable voxel-wise reference labels are unavailable, RSD serves as the primary label-free evaluation criterion. Taken together, the HCP and CNP RSD analyses provide a common homogeneity-based perspective across datasets, but they do not replace the need for future supervised validation on external datasets with reliable diffusion-space DK reference labels.

Supplementary Note 6: Voxel-wise uncertainty analysis

To further assess the robustness of the predicted parcellations, we performed a post-hoc uncertainty analysis based on the softmax output probabilities.

For each voxel, we compute three standard measures:

- **Confidence**: the maximum class probability,

$$\text{confidence} = \max_c p_c$$

- **Uncertainty**: defined as

$$\text{uncertainty} = 1 - \text{confidence}$$

- **Margin**: difference between the top two class probabilities,

$$\text{margin} = p_{(1)} - p_{(2)}$$

where p_c denotes the predicted probability for class c, and $p_{(1)}$ and $p_{(2)}$ are the highest and second-highest probabilities, respectively.

Qualitative analysis Supplementary Figure 1 shows representative slices comparing nnUNet and our method. As expected, uncertainty, reflected by low confidence and low margin, is primarily concentrated near anatomical boundaries. Compared to nnUNet, our method exhibits better confidence.

Quantitative summary We further compute the mean values of these measures within the brain mask.

- $OURS_{unet}$: confidence = 0.951, uncertainty = 0.049, margin = 0.906
- nnUNet: confidence = 0.909, uncertainty = 0.091, margin = 0.828

These results indicate that our method produces higher-confidence predictions with lower uncertainty. We emphasize that this uncertainty analysis is not used during training and is provided as an additional robustness indicator.

Supplementary Table 6. Cost-benefit comparison between the standalone single-stage models and the corresponding hierarchical versions of our framework. Training cost is reported in GPU hours using the same hardware and training setup. Inference time is reported as average wall-clock time per subject.

Backbone	DSC	Training GPU hours	Inference (sequential, s)	Inference (parallel, s)
nnUNet	79.64	48.08	2.58	–
$OURS_{unet}$	81.12	159.65	9.45	3.18
MedNeXt-M-K3	81.35	80.67	9.46	–
$OURS_{MedNeXt}$	82.09	239.98	31.44	10.66
SwinUNETR	79.05	65.52	9.90	–
$OURS_{SwinUNETR}$	79.92	210.03	55.73	18.70

Supplementary Note 7: Cost-benefit analysis of the hierarchical framework

To quantify the computational trade-off of the proposed hierarchical framework, we compared each standalone backbone with its corresponding hierarchical version. The standalone nnUNet, MedNeXt-M-K3, and SwinUNETR models serve as the single-stage baselines, whereas $OURS_{unet}$, $OURS_{MedNeXt}$, and $OURS_{SwinUNETR}$ denote the corresponding two-stage versions composed of one coarse model followed by five fine subnetworks.

Training cost was measured in GPU hours using the same hardware and implementation settings as in the main experiments. For the hierarchical models, the total training cost was computed as the sum of the coarse model and all fine subnetworks. Inference time was measured per subject for both sequential execution and parallel execution of the fine subnetworks.

Importantly, although the hierarchical framework contains one coarse model and five fine subnetworks, the computational overhead is substantially lower than a naive sixfold increase. This is because the fine subnetworks solve smaller compartment-specific refinement tasks rather than the full 101-label segmentation problem. As a result, the increase in training cost is approximately 3.32×, 2.97×, and 3.21× for the U-Net, MedNeXt, and SwinUNETR backbones, respectively, rather than 6×. Specifically, the training cost increased from 48.08 to 159.65 GPU hours for the U-Net backbone, from 80.67 to 239.98 GPU hours for MedNeXt, and from 65.52 to 210.03 GPU hours for SwinUNETR. Sequential inference time increased from 2.58 to 9.45 seconds for U-Net, from 9.46 to 31.44 seconds for MedNeXt, and from 9.90 to 55.73 seconds for SwinUNETR. However,

because the five fine subnetworks are independent once the coarse prediction is available, they can be executed in parallel. Under parallel execution, the wall-clock inference time was reduced to 3.18 seconds for U-Net, 10.66 seconds for MedNeXt, and 18.70 seconds for SwinUNETR, corresponding to approximately 1.23×, 1.13×, and 1.89× the single-stage baselines.

These additional costs are accompanied by consistent accuracy gains. Relative to the corresponding standalone models, the hierarchical framework improves DSC by 1.48 percentage points for U-Net, 0.74 percentage points for MedNeXt, and 0.87 percentage points for SwinUNETR. Overall, these results indicate a favorable cost-benefit trade-off, with computational overhead that is substantially below the number of subnetworks involved and accompanied by consistent improvements in segmentation accuracy.
